# Situations restructure the congruency between action and valence in the action-evaluation effect

**DOI:** 10.1038/s41598-018-23095-x

**Published:** 2018-03-20

**Authors:** Hanlin Wang, Jiushu Xie, Ce Mo, Xianyou He, Ruiming Wang, Rongjun Yu, Lei Mo

**Affiliations:** 1 Center for Studies of Psychological Application, South China Normal University, Guangzhou, China; 2 School of Education, Hebei Normal University, Shijiazhuang, China; 3 School of Psychology, South China Normal University, Guangzhou, China; 4 Guangdong Provincial Key Laboratory of Mental Health and Cognitive Science, South China Normal University, Guangzhou, China; 5 Peking-Tsinghua Center for Life Sciences, Academy for Advanced Interdisciplinary Studies, Peking University, Beijing, China

## Abstract

The action-evaluation effect indicates that the processing of affective valence is affected by bodily actions. However, whether this effect is based on bodily simulation or situational priming is unknown. Moreover, P2 is a neural marker for this effect, suggesting the integration between valence and actions. Whether the P2 component is modulated by the situation is also unknown. In this study, we tested this effect in multiple situations to examine (1) whether this effect is dependent on the situation and (2) the amplitude of P2 is modulated by the situation. During the experiments, participants pushed/pulled computer mice to verify the valence of affective words in far-near (Experiment 1), front-back (Experiments 2a-2b), and up-down (Experiments 3a-3b) situations. Pulling (or pushing) mice responding to positive (or negative) words were treated as the congruent condition, while the opposite combination was the incongruent condition. In the far-near situation, participants’ response times were faster and the amplitude of the P2 component was smaller in the congruent condition than the incongruent one; however, these results were reversed in other situations. The results suggested that the congruency of action-evaluation effect was restructured by the situation. Therefore, the action-evaluation effect might be based on situational priming.

## Introduction

Previous findings on embodied cognition show that bodily actions play a key role in human cognition^1–8^. For example, participants respond more quickly to positive and negative words by pulling and pushing levers, respectively, which is known as the action-evaluation (embodiment) effect and also as the approach/avoidance effect^9–12^.

The action-evaluation effect has been well documented. Chen and Bargh^9^ note that people tend to non-consciously classify stimuli as positive and negative. This automatic evaluation would result in behavioural predispositions. For example, positive and negative evaluations would result in approach and avoidance tendencies, respectively. These tendencies have been tested in the far-near situation: participants push away something from themselves (far) or pull something towards themselves (near). The results showed that participants’ responses were faster when they pushed mice (avoid) to respond to negative words and pulled mice (approach) to respond to positive words.

Regarding the action-evaluation effect, embodied cognition interprets that evaluating affective stimuli through pushing and pulling actions automatically triggers muscle contraction/diastole or previous sensory/motor information^9–12^. Sensory/motor information indicates memories about sensing the environment and bodily states through sense organs.

Based on this interpretation, the action-evaluation effect may be derived from two mechanisms: bodily simulation and situational priming. First, the bodily simulation mechanism suggests that the processing of affective concepts is grounded in sensorimotor experience. When one processes an affective concept, related muscle contractions and skeletal motions are activated^5^. Thus, bodily actions are essential for conceptual processing. Most studies have adopted this approach to interpret their findings^13–15^. Moreover, this account also holds that the action-evaluation effect shows a constant association because people already build conditioned associations between arm flexion and approach and between arm extension and avoidance^16^. Similar effects have also been found in associations between dominant hands and positivity and between non-dominant hands and negativity^17,18^.

Second, however, the action-evaluation effect may also be interpreted by an experimental priming in a specific situation derived from individuals’ previous experiences, which is known as situational priming. People usually avoid negative stimuli and approach positive stimuli. Negative stimuli are associated with pushing actions and positive stimuli with pulling actions. These associations between bodily actions and valence are stored in one’s long-term memory and activated when affective information is presented. In other words, the processing of affective words first activates the stored association between bodily action and valence (i.e., previous experiences) and then induces the embodied phenomenon. Specifically, when participants push/pull a lever, corresponding affective experiences in similar situations related to valence information are primed and then modulate their responses. Thus, the congruency effect in the action-evaluation effect is induced by situational priming.

However, embodied processes cannot occur in a vacuum. How then do situational settings (i.e., the context in which embodied processes occur) interact with bodily actions to modulate the action-evaluation effect? Based on the situational priming account, as the same action usually associates with different valences under diverse situations, the action-evaluation effect may accordingly also be stored in multiple forms in one’s long-term memory. For example, when one holds a positive object, pulling a hand is more adaptive than pushing a hand; when one holds a negative object, pushing a hand is more adaptive than pulling a hand. Hence, the action-evaluation effect should be flexible, but not constant, according to the situational priming hypothesis, which cannot be predicted by the bodily simulation account.

Previous studies have also provided initial evidence supporting the situational priming account. For example, a reference (e.g., a block presenting a famous person’s photo) presented in the background of the valence judgement task modulated the action-evaluation effect: when the referenced photo was positive, participants were faster to move positive words towards and negative words away from the photo than the opposite actions; when the photo was negative, participants’ performance showed a reversed pattern^19^. Moreover, several studies have found that through conditioned training, the execution of neutral actions (e.g., key pressing) can exhibit effects similar to the action-evaluation effect^20–22^. These findings suggest that the action-valence association may be malleable and can be conditioned. However, previous studies did not test the inner mechanisms of this association. Hence, whether this malleable association is a special phenomenon induced by some experimental manipulation or a universal phenomenon induced by situational priming remains unknown. The malleability of the action-valence association is important evidence to distinguish situational priming from bodily simulation. To verify these two alternative explanations, we should manipulate situational settings, in which the action-evaluation effect occurs, to examine whether the effect is modulated by situations.

According to the initial evidence, although pulling/pushing adopts constant muscle contraction/diastole, the action-evaluation effect may be restructured by situations. This influence cannot be predicted by the bodily simulation account. Therefore, we hypothesize that this association is not merely driven by bodily actions but is also based on a situational priming hypothesis. When people process affective information in specific situations, previous experiences between affective information and actions in such situations will be activated and then modulate the processing of affective valence before bodily actions.

If our hypothesis is correct, we predict that the same bodily actions will induce various action-evaluation effects under different situations. Therefore, we tested action-evaluation effects in far-near (Experiment 1), front-back (Experiments 2a and 2b), and up-down (Experiments 3a and 3b) situations to examine the potential situation-dependency of such effects. In each situational setting, participants push or pull a computer mouse to execute the same pushing and pulling actions to respond to affective words. Specifically, participants’ pushing actions can be interpreted as moving something far away from themselves (Experiment 1), moving forward (Experiments 2a and 2b), and moving upward (Experiments 3a and 3b); their pulling actions can be interpreted as moving something close to themselves (Experiment 1), moving backward (Experiments 2a and 2b), and moving downward (Experiments 3a and 3b). Thus, the same actions can be interpreted by different situational experiences in different situations. If action evaluation effects were based on situational priming, combinations between pushing/pulling and positive/negative words would be varied under different situations. For example, in Experiment 1, pulling actions would be associated with positive valence and pushing actions with negative valence. In contrast, this association would be reversed in Experiments 2 and 3. According to previous studies, we define push negative words (and pull positive words) as the congruent condition and pull negative words (and push positive words) as the incongruent condition. To help readers better understand results, all experiments reported herein adopted this definition.

Moreover, the event-related potentials (ERP) technique was adopted in the present study to examine whether situational settings modulated the processing of affective words. We focused on the P2 component that peaks approximately 200 ms post-stimuli presentation, which has been reported as the index for integrating multidimensional information of stimuli in previous studies^12,23,24^. According to Wang, *et al*.^12^, who used a paradigm similar to the one in the present study, when bodily actions are congruent with affective valence, P2 is smaller when they are incongruent. We also predicted that the congruency between affective words and bodily actions would be restructured by different situations. Therefore, we focused on how the amplitude of the P2 component is modulated by the congruency in the action-evaluation effects across different situations, while we have already pre-defined congruent and incongruent conditions in all experiments.

## Results

### Experiment 1 (the far-near situation)

In Experiment 1, we conceptually replicated the classical action-evaluation effect in a far-near situation (i.e., push-away-bad/pull-towards-good). Participants pushed or pulled the mouse to verify an affective word that appeared at the centre of the screen. The mouse was placed on the participants’ right frontal sides.

**Figure 1:**
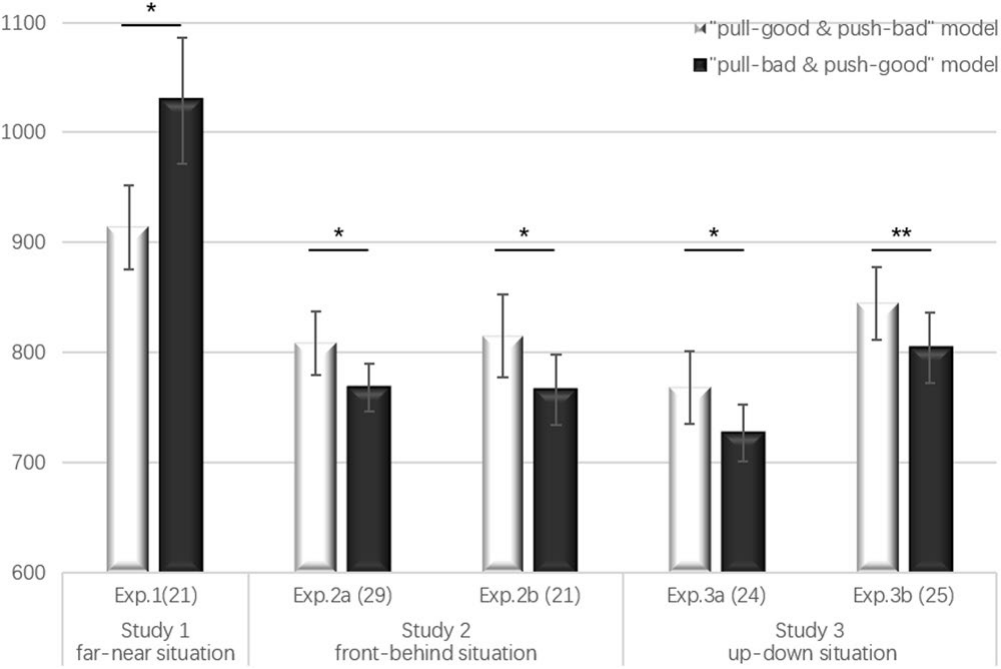
RTs (in ms) to affective words as a function of bodily action in five experiments (error bars indicate standard errors, *indicates *p* < 0.05; **indicates *p* < 0.01).

The behavioural results of Experiment 1 showed that participants evaluated affective words faster in the congruent condition (mean ± *SE*, 914 ± 38 ms) than in the incongruent condition (1029 ± 57 ms), *t*(20) = −2.598, *p* = 0.017 (two-tailed), 95% CI [−207.353, −22.664], *d* = −0.518, replicating the classic action-evaluation effect (see Table 1 and Fig. 1).

**Table 1:** Mean response times (RTs, in milliseconds) for all four conditions with standard deviations (*SD*) in the three situations.

		Negative-Pull	Negative-Push	Positive-Pull	Positive-Push
Far-Near	Experiment 1	1056 ± 295	929 ± 166	901 ± 192	1004 ± 238
Front-Behind	Experiment 2a	796 ± 108	813 ± 173	801 ± 147	740 ± 134
	Experiment 2b	784 ± 140	834 ± 196	786 ± 148	737 ± 153
Up-Down	Experiment 3a	750 ± 131	771 ± 163	765 ± 170	706 ± 130
	Experiment 3b	826 ± 172	857 ± 175	832 ± 160	781 ± 147

**Figure 2:**
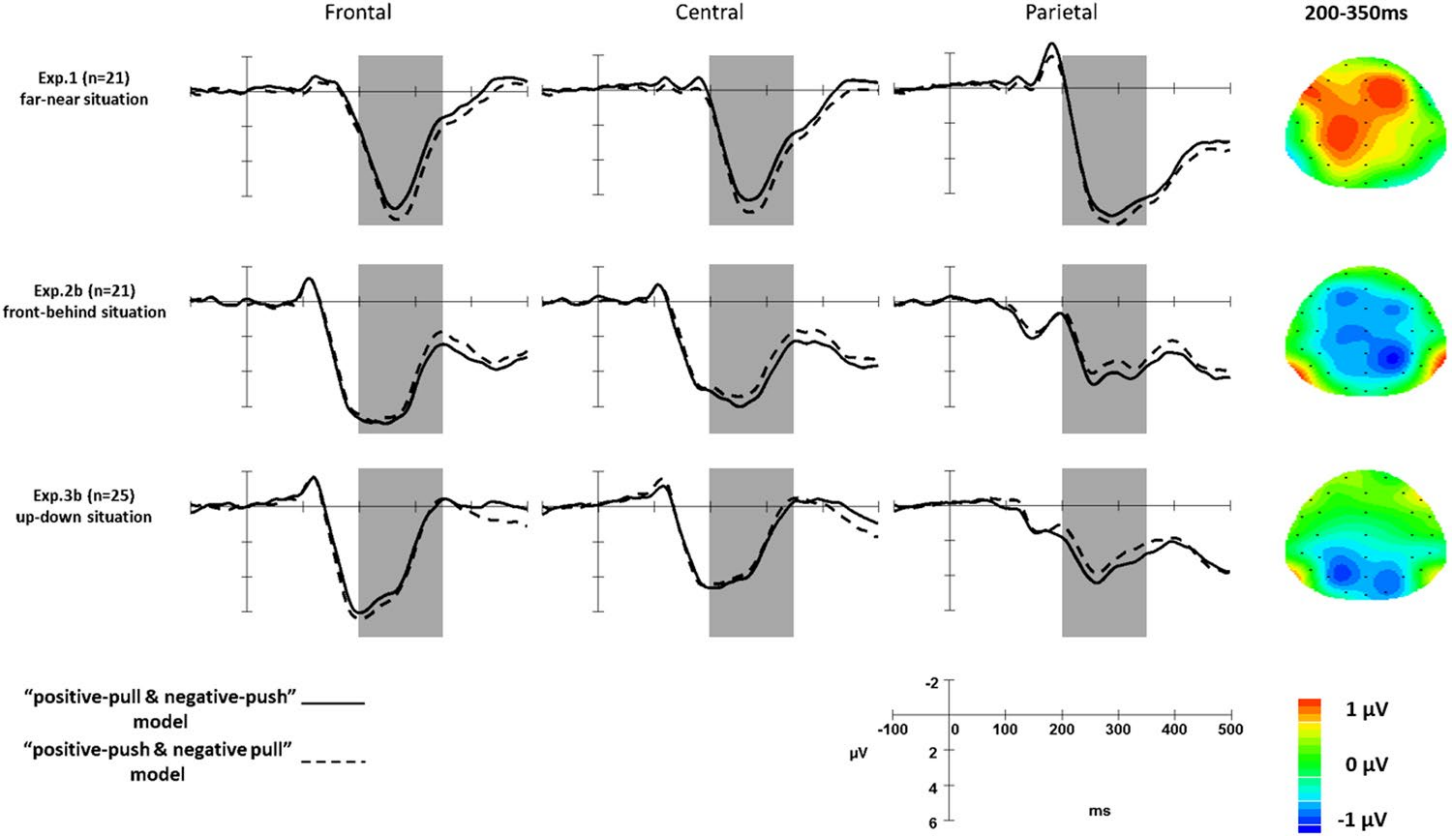
Grand-average amplitudes of the stimulus-locked ERPs at frontal, central, and parietal areas (left) with topographic voltage scalp maps (right) of the P2. The difference-wave topography showed the grand amplitude of the P2 within 200–350 ms, which was calculated by subtracting the amplitude value of the solid line from the dotted line. Grey rectangles indicate the analysis window (i.e., 200–350 ms) for the P2 component.

**Figure 3:**
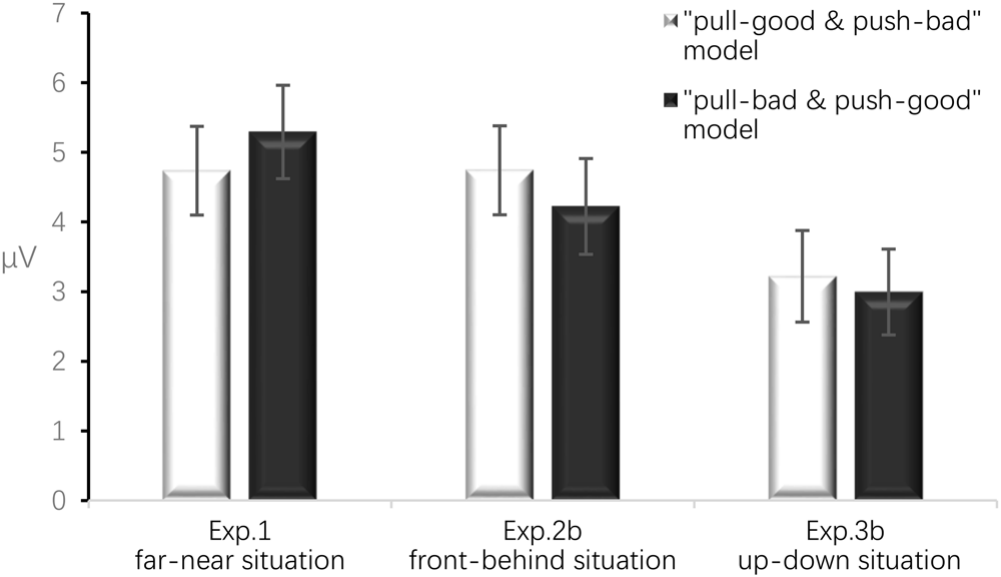
Mean amplitude of the P2 (μV) across conditions (error bars indicate standard errors).

All ERP results reported herein focus on the mean amplitude of the P2 component between 200 and 350 ms after the presentation of the affective words. A 2 (condition: congruent vs. incongruent) × 5 (electrode areas: frontal, frontal-central, central, central-parietal, and partial) repeated-measure ANOVA showed a significant main effect of condition, *F*(1,20) = 5.590, *p* = 0.028, 95% CI [0.066, 1.049], *η*_*p*_^2^ = 0.218. Importantly, the incongruent condition (5.292 ± 0.670 μV) induced a larger P2 potential than the congruent condition (4.735 ± 0.637 μV). In addition, the main effect of the electrode areas showed a marginal trend towards significance, *F*(4,80) = 3.602, *p* = 0.054, *η*_*p*_^2^ = 0.153. The interaction between condition and electrode areas was not statistically significant, *F*(4,80) = 0.836, *p* = 0.397 (see Table 2, Figs 2 and 3).

**Table 2:** Mean amplitude of the P2 (μV) with standard errors (*SE*) in the “far-near” situation.

	Negative		Positive	
	Pull	Push	Pull	Push
Frontal	5.12 ± 0.67	4.34 ± 0.6	4.52 ± 0.78	4.95 ± 0.71
Frontal-Central	5.04 ± 0.75	4.23 ± 0.66	4.41 ± 0.82	4.93 ± 0.78
Central	4.97 ± 0.73	4.27 ± 0.65	4.48 ± 0.76	4.92 ± 0.78
Central-Parietal	5.45 ± 0.67	5.02 ± 0.63	4.95 ± 0.7	5.52 ± 0.72
Parietal	5.8 ± 0.68	5.64 ± 0.72	5.36 ± 0.73	5.9 ± 0.72

This experiment conceptually replicated previous findings on the action-evaluation effect, indicating that the current paradigm was valid. In the following experiments, the situations are directly manipulated to test whether similar bodily actions elicit different action-evaluation effects under different situations.

### Experiments 2a and 2b (the front-back situation)

The results of Experiment 2a indicate that participants evaluated affective words faster in the incongruent condition (768 ± 22 ms) than in the congruent condition (808 ± 29 ms), *t*(28) = −2.352, *p* = 0.026 (two-tailed), 95% CI [−74.631, −5.151], *d* = −0.286 (see Table 1 and Fig. 1).

The behavioural results of Experiment 2b also showed the same pattern as that in Experiment 2a between the incongruent (766 ± 32 ms) and congruent conditions (815 ± 38 ms), *t*(20) = −2.460, *p* = 0.023 (two-tailed), 95% CI [−90.468, −7.437], *d* = −0.303 (see Table 1 and Fig. 1). The 2 (condition: congruent vs. incongruent) ×5 (electrode areas: frontal, frontal-central, central, central-parietal, and partial) repeated-measure ANOVA of the ERP results showed a significant main effect of condition, *F*(1, 20) = 7.959, *p* = 0.011, 95% CI [0.135, 0.901], *η*_*p*_^2^ = 0.285. More importantly, the congruent condition (4.742 ± 0.638 μV) elicited a larger P2 potential than the incongruent one (4.223 ± 0.689 μV). The main effect of electrode areas was significant, *F*(4, 80) = 6.054, *p* = 0.017, *η*_*p*_^2^ = 0.232. However, post hoc tests showed that all comparisons were not significant when Bonferroni correction was used. The interaction between condition and electrode areas was not statistically significant, *F*(4, 80) = 0.387, *p* = 0.620 (see Table 3, Figs 2 and 3). These results are the opposite of those obtained in Experiment 1, indicating that in the front-back situation, participants associated positive and negative words with push and pull, respectively, and supporting our situational priming hypotheses.

**Table 3:** Mean amplitude of P2 (μV) with standard errors (*SE*) in the “front-behind” situation.

	Negative		Positive	
	Pull	Push	Pull	Push
Frontal	3.68 ± 0.73	3.8 ± 0.71	3.98 ± 0.57	3.55 ± 0.72
Frontal-Central	3.38 ± 0.77	3.67 ± 0.7	3.83 ± 0.62	3.31 ± 0.71
Central	2.92 ± 0.72	3.3 ± 0.64	3.17 ± 0.63	2.75 ± 0.68
Central-Parietal	2.51 ± 0.71	2.82 ± 0.62	2.88 ± 0.69	2.29 ± 0.69
Parietal	1.96 ± 0.76	2.21 ± 0.75	2.1 ± 0.77	1.65 ± 0.75

### Experiments 3a and 3b (the up-down situation)

The results of Experiment 3a also showed that participants’ responses were faster in the incongruent condition (727 ± 26 ms) than in the congruent condition (768 ± 33 ms), *t*(23) = 2.290, *p* = 0.032 (two-tailed), 95% CI [3.921, 77.235], *d* = −0.280 (see Table 1 and Fig. 1).

The behavioural results of Experiment 3b showed the same results as those in Experiment 3a between the incongruent (804 ± 32 ms) and congruent conditions (845 ± 33 ms), *t*(24) = −3.170, *p* = 0.004 (two-tailed), 95% CI [−67.196, −14.206], *d* = −0.26 (see Table 1 and Fig. 1). The 2 (condition: congruent vs. incongruent) × 5 (electrode areas: frontal, frontal-central, central, central-parietal, and partial) repeated-measure ANOVA of the ERP results showed a significant interaction between condition and electrode areas, *F*(4, 96) = 8.165, *p* = 0.005, *η*_*p*_^2^ = 0.254. Critically, simple effects tests using *F*-tests and Bonferroni correction showed that in the central-parietal and parietal areas, the congruent condition induced a larger P2 than the incongruent one, *p* < 0.05 (see Table 4, Figs 2 and 3). The present results also revealed an opposite “push-up-good and pull-down-bad” association compared with the association in Experiment 1.

**Table 4:** Mean amplitude of P2 (μV) with standard errors (*SE*) in the “up-down” situation.

	Negative		Positive	
	Pull	Push	Pull	Push
Frontal	3.7 ± 0.74	3.26 ± 0.82	3.61 ± 0.86	3.63 ± 0.68
Frontal-Central	3.08 ± 0.74	2.84 ± 0.82	3.22 ± 0.83	3.08 ± 0.69
Central	2.64 ± 0.71	2.74 ± 0.7	3.05 ± 0.7	2.65 ± 0.63
Central-Parietal	2.84 ± 0.64	3.27 ± 0.6	3.34 ± 0.64	2.77 ± 0.6
Parietal	2.81 ± 0.59	3.42 ± 0.56	3.45 ± 0.61	2.74 ± 0.57

By combining the results of the three experiments, the action-evaluation effect was modulated by situations. In the far-near situation, pulling and pushing actions might trigger approaching and avoiding experiences, respectively; in the front-back and up-down situations, pulling and pushing actions might trigger avoiding and approaching experiences, respectively. Thus, the action-evaluation effect showed diverse combinations under different situations.

## Discussion

We conducted 3 experiments to test the situation dependence of the action-evaluation effect in distinct situations. Participants pushed or pulled a computer mouse to respond to the valence of affective words (i.e., positive or negative) in all 3 experiments. In Experiment 1, in a far-near situation, a larger P2 component and slower response times were found when participants pushed positive words and pulled negative words than when their responses were reversed. In Experiment 2, in a front-back situation, a larger P2 component and slower response times were found when participants pushed negative words and pulled positive words than when their responses were reversed, showing an opposite combination pattern with Experiment 1. In Experiment 3, in an up-down situation, a larger P2 component and slower response times were found when participants pushed negative words and pulled positive words than when their responses were reversed, showing an opposite combination pattern with Experiment 1. In short, we found that the action-evaluation effect was modulated by situations: combinations between valence and bodily actions were varied in different situations, supporting the situational priming account. In addition, the results also confirmed that the P2 component was a valid ERP index for the action-evaluation effect, which appeared approximately 200 ms after the onset of affective words.

The present findings support the situational priming account. In 3 different situations, participants executed the same bodily actions but elicited distinct action-evaluation effects. The association between action and valence is modulated by situations. In the far-near situation, pushing is interpreted by push-avoid associated with negative valence and pulling is interpreted by pull-approach associated with positive valence. In contrast, in front-back and up-down situations, pushing is defined as push-front and push-up, respectively, for which positive labels are assigned, whereas pulling is defined as pull-back and pull-down, respectively, for which negative labels are assigned. Thus, although the same push-pull actions are executed in different situations, these actions are accompanied by different action-evaluation effects, which cannot be predicted by the bodily simulation account.

In this case, bodily actions might be induced by situational priming. When participants verified an affective word, their experiences related to this word, such as approaching or avoiding something, were also activated. Thus, this activation would affect participants’ bodily actions and elicit action-evaluations effects.

The present study revealed that the discrepancy between congruent and incongruent associations between actions and affective valence emerged approximately 200 ms after the presentation of affective stimuli. This discrepancy is reflected in the amplitude of the P2 component, the first ERP component in which divergence appeared. We have also analysed other components that appeared before P2, such as N1. However, the N1 component did not reflect any interesting divergences regarding the present research topic. For detailed results of N1, please see Table S3 in the supplementary material.

The P2 component has been shown to be an index for integrating information across sensory modalities^23–25^. Previous studies have used ERP technology to test the integration of grapheme and colour. The results indicated that when the integration became difficult, the amplitude of the P2 was enhanced^26^. In the present study, to complete experimental tasks, participants executed pushing or pulling actions according to the affective valence. Embodied cognition holds that affective valence is interpreted by sensory/motor information, such as pushing or pulling actions^2,4,6,8,27^. When a participant watches a positive word, his/her previous situational experiences when s/he interacted with positive valence are activated (e.g., pull his/her favourite toys towards him/herself)^17,28–30^. These activated experiences induced by affective valence are integrated with the pushing/pulling actions. When participants’ present bodily actions are congruent with the affective valence according to situational experiences, the integration between bodily actions and affective words is facilitated compared with incongruent conditions. Specifically, response times are faster and the amplitude of P2 is smaller in the integration of congruent compared with incongruent conditions.

Although the effect of P2 in the present study might be interpreted as the index of integrating information, there are two potential alternative interpretations of the P2 effect. (1) The P2 effect may reflect the change in attentional load. Previous studies have shown that P2 can be affected by attention recruitment. When stimuli attract or elicit attention resources, the amplitude of the P2 component becomes larger^31–34^. In the present study, participants had to push or pull mice to respond to affective words. When the actions were incompatible with affective valence (e.g., pushing a positive word in the far-near situation), participants had to allocate more attention resources to complete this trial. (2) Likewise, when participants were asked to complete an incompatible action (e.g., pushing a positive word in the far-near situation), more inhibition was required to inhibit the compatible action (e.g., pulling a positive word in the far-near situation). Therefore, the enhanced attention load and inhabitation in incompatible conditions might also contribute to the P2 effect. Further studies are needed to examine these alternative interpretations.

The present ERP results are similar to previous findings. Wang, *et al*.^12^ adopted a similar paradigm to test how distance modulates the action-evaluation effect in the far-near situation. In that experiment, participants also pushed or pulled mice to respond to affective words. The ERP results showed that the amplitude of the P2 component in the congruent condition (positive-pulling and negative-pushing) was smaller than that in the incongruent condition (positive-pushing and negative-pulling). We also conceptually replicated this finding in our Experiment 1, indicating that our paradigm was valid. Thus, the incongruent integration of the action-evaluation effect elicited an enhanced P2 component and slower response times. In Experiments 2 and 3, if the action-evaluation effect was independent of the situation, we would still find an enhanced P2 component and slower response times when participants pushed positive and pulled negative words. However, the results of Experiments 2 and 3 showed opposite patterns. In the front-back and up-down situations, the incongruent integrations were pulling-positive and pushing-negative. Hence, the congruency of the action-evaluation effect was restructured, and this effect mainly based on situational priming.

The P2 component has also been reported in previous studies on morality- and valence-space metaphors^35,36^. In the valence-space metaphor study, participants first remembered some affective words and then completed a spatial cue detection task, in which a white dot appeared at the top or bottom of the screen. The results indicated that the top cues induced an enhanced P2 component when participants kept positive rather than negative words in mind. Bottom cues induced a reserved effect^35^. However, the present study found that the P2 component increased in the incongruent rather than the congruent condition (Experiment 1), even though P2 is a valid index for both of them. This phenomenon may be induced by the different paradigms adopted in these experiments. The valence-space metaphor study adopted a cuing paradigm and presented primes and probes separately, while the present study presented them simultaneously. Kanske, *et al*.^37^ revealed that the P2 component was enhanced by emotionally valid rather than invalid cues. This P2 component might reflect emotional orientation, i.e., emotional cues (e.g., affective words) boost attention allocation, which was reflected by an enlarged P2 component. This result is also in agreement with the well-documented interpretation of the spatial attention effects reflected by early ERP components^38^. Thus, the P2 component revealed by Xie, *et al*.^35^ reflected the spatial distribution of attention. In contrast, in the present study, participants responded to affective words as soon as they appeared. Participants did not need to distribute attentional resource at different positions. Similarity, Wang, *et al*.^36^ also presented affective words and location information simultaneously. P2 in both studies may not reflect attention distribution, which may explain why the incongruent integration condition elicited a larger P2 in the present analysis.

Further studies combining ERP technology and electromyographic recording to test the time course of the P2 component and muscle activities (or action preparation) would reveal whether involuntary muscle activities are indispensable for the action-evaluation effect.

The action-evaluation effect could be interpreted by the approaching and avoiding motivation. Embodied cognition suggests that pushing and pulling actions automatically trigger avoidance or approach feelings, thus influencing the evaluation of the valence stimulus^16,21,39,40^. Chen and Bargh^9^ state that people always unconsciously divide stimuli into positive and negative. Positive and negative evaluations would induce approach and avoidance tendencies, respectively. Thus, in Experiment 1’s far-near situation, participants’ performance was better when they pushed mice (avoid) to respond to negative words and pulled mice (approach) to respond to positive words. In contrast, in Experiment 2’s front-back situation, pushed and pulled mice could be interpreted as moving forward and backward, respectively. Usually, people move forward in line with their facing direction. Thus, pushing is interpreted as approach and pulling is interpreted as avoidance. Similarly, in Experiment 3’s up-down situation, pushed and pulling mice could be interpreted as raising up and going down, respectively. While people usually associate good with up and bad with down, such as happy is up and sad is down, people would like to approach upper locations and avoid lower ones^41,42^. Thus, pushing up is interpreted as approach and pulling down is interpreted as avoidance^43^. Together, pushing and pulling actions triggered approach and avoidance motivations and then affected participants’ response to affective words^12^. The association between push/pull actions and approach/avoidance motivations is modulated by the situation.

Above all, the present findings showed that situational settings modulated the action-evaluation effect, suggesting that this effect was based mainly on situational priming, not on bodily simulation. Furthermore, this situational priming was reflected by the P2 component. In short, the present study revealed that situations restructured the congruency between bodily actions and affective valence in the action-evaluation effect supporting the situational priming account.

## Methods

### Experiment 1 (the far-near situation)

#### Participants

Twenty-five healthy undergraduate students were recruited randomly for Experiment 1; four of them were excluded from data analysis due to excessive artefacts or anomalies in the electroencephalography (EEG) signals. In the end, data from 21 participants (10 males, mean ± *SE*, 22 years ± 2.1) were included in final analyses. The number of participants in this and the following experiments were based on a previous study adopting the same paradigm^12^. All participants were right-handed, were native speakers of Chinese, and had normal or corrected-to-normal vision. They provided written informed consent before the experiment. The experiment was approved by the ethical review board of the Department of Psychology, South China Normal University. All methods used in the current study were performed in accordance with the relevant guidelines and regulations of the ethical review board.

#### Materials and design

One hundred and sixty Chinese affective words were selected from a public affective word database, in which the valence of each word was rated on a 9-point scale (1 is extremely negative; 9 is extremely positive)^44^. All words contained two Chinese characters. Half of them were positive, and half were negative. Mean affective valences for positive words were 6.94 ± 1.31 and for negative words 2.97 ± 1.30. None of the words’ semantic meanings were related to distance, location, or motion, which were independent variables in this work.

To investigate participants’ subjective evaluations of the associations between affective words and bodily actions along the far-near direction, we conducted a separate rating prior to the critical experiment. We recruited another 30 undergraduate students and selected another 20 affective words to use in this rating. The students completed trials in which they pushed and pulled computer mice to respond to positive and negative words, respectively. In all subjective evaluations in the current study, all participants’ postures and actions were identical to the postures and actions in the main experiment. Next, they answered a multiple-choice question to evaluate their preference for the combined responses between affective words and bodily actions. The question was as follows: When you were performing the pushing and pulling actions, which combinations below best describe your feelings aroused by push/pull actions? The choices were (1) push-avoidance and pull-approach; (2) push-front and pull-back; (3) push-up and pull-down; (4) other; (5) do not have any particular feelings. The results showed that 27 participants (90%) chose (1), 2 participants (6.7%) chose (2), and 1 participant chose (5), suggesting that the majority of the participants preferred the “push-avoidance and pull-approach” combination.

The experiment adopted a single factor within-subject design: the congruency between affective words and bodily actions (congruent vs. incongruent). The congruent condition indicated that participants pulled (or pushed) computer mice to respond to positive (or negative) words; the incongruent condition indicated that participants pushed (or pulled) computer mice to respond to positive (or negative) words.

#### Procedure

**Figure 4:**
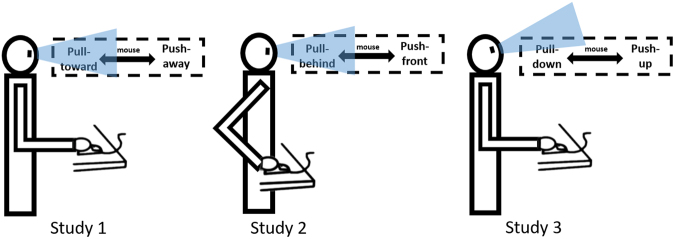
An illustration of participants’ actions in Experiments 1–3.

**Figure 5:**
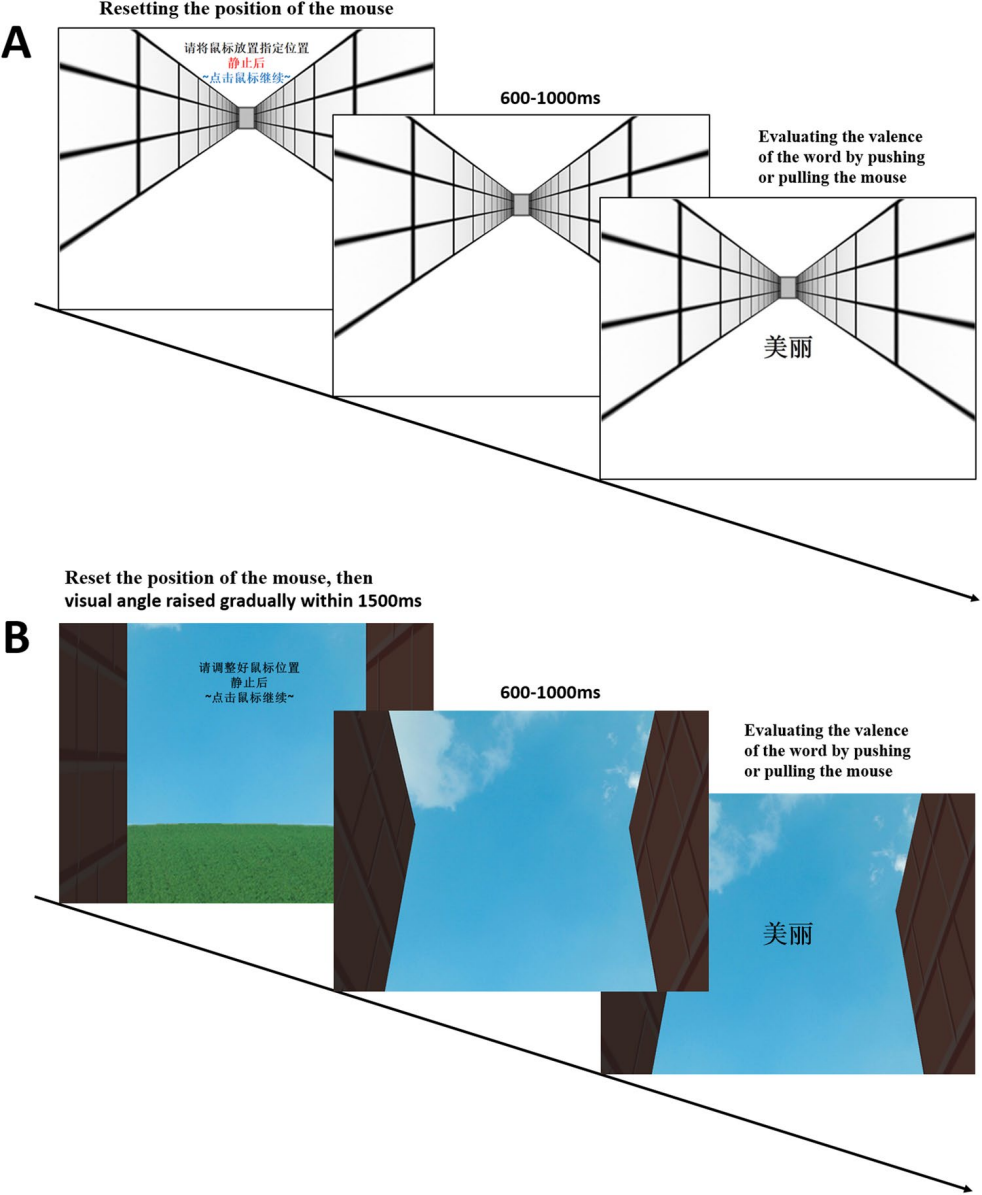
Sequences of events for a trial in Experiments 1, 2a, and 2b (Panel A) and Experiments 3a and 3b (Panel B). The Chinese affective word “” on the last slides of Panels A and B means “beauty.”

Participants sat approximately 70 cm from the display in a comfortable position. Their right hands held a computer mouse at the right frontal sides of their bodies. The initial hand position was marked on the desk. The resolution of the display was 640 × 480 pixels. In the experiment, stimuli were presented on a corridor that produced an illusion of depth (see Figs 4 and 5, Panel A).

At the beginning of each trial, after a random duration of 600–1000 ms, a target word was presented in the centre of the corridor in 30-point font waiting for participants’ responses. Simultaneously, an invisible cursor was automatically reset to the centre of the screen (X = 320 pixels, Y = 240 pixels). Participants had to verify the valence of the affective word by pushing or pulling the mouse. The invisible cursor moved together with the mouse from the reset location (Y = 240 pixels) and terminated when the cursor moved to the top boundary (Y = 30 pixels) or the bottom boundary (Y = 450 pixels). After the invisible cursor reached the upper or lower border of the screen, the target word disappeared. Participants’ response times were recorded from the appearance of affective words to the time when the cursor moved to the boundary. After 500 ms, the next trial began. In the congruent condition, participants were instructed to pull the mouse towards themselves for positive words and push the mouse away for negative words. In the incongruent condition, participants were instructed to push the mouse away for positive words and pull it towards themselves for negative words. No participants were aware of a special association between these actions and valences.

Since action would induce muscle artefacts during the EEG recording, mouse sensitivity was set at a higher value and participants’ wrists were fixed on a soft pad to minimize the influence of muscle artefacts. The participants were required to stretch their fingers to execute pushing or pulling actions. Thus, the trajectory of the mouse’s action was approximately 3 cm for both the pushing and pulling actions.

Participants completed congruent and incongruent blocks sequentially. Each block contained 160 randomly ordered trials (80 trials with positive words and another 80 trials with negative words). The order of these two blocks was counter-balanced between participants. A practice session was included before the experiment.

#### EEG recording and pre-analysis

We recorded EEG signals from 32 electrode sites (according to the international 10–20 system) using a Neuroscan system (Neuroscan Inc., Herndon, VA, USA). Recorded electrodes were referenced to the left mastoid online and were re-referenced to the average of the left and right mastoids off-line. Bipolar horizontal and vertical EOG were recorded simultaneously to monitor eye actions. Impedances of all electrodes were maintained at lower than 5 kΩ. The sampling rate was 1000 Hz. The data were filtered online using a 0.05–100 Hz bandpass filter and were re-filtered offline with a 30-Hz low pass filter.

Ocular artefacts were corrected with an eye-action correction algorithm used in Scan 4.5 (Charlotte, NC, USA)^45^. ERP were time-locked to the onset of the presentation of the target word. Epochs ranged from −100 to 500 ms after the onset of the stimulus. The 100-ms interval preceding the stimulus onset served as the baseline. A criterion of ±80 μV was used to reject artefacts. ERP were assessed via mean amplitude measurements of the P2 component between 200–350 ms after the presentation of affective words. Mean amplitudes of the P2 component were submitted to a 2 (condition: congruent vs. incongruent) ×5 (electrode areas: frontal, frontal-central, central, central-parietal, and partial) repeated measures of analysis of variance (ANOVA). We divided these electrode sites into five areas: frontal (F3, Fz, F4); frontal-central (FC3, FCz, FC4); central (C3, Cz, C4); central-parietal (CP3, CPz, CP4); and partial (P3, Pz, P4). A Greenhouse-Geisser correction was applied when necessary. Incorrect trials and trials whose response times were outside ±2.5 SDs from the participants’ mean response time were excluded from the analyses (6.2%).

### Experiments 2a and 2b (the front-back situation)

To further examine hypotheses regarding the situational priming account for the action-evaluation effect, we conducted Experiments 2 and 3 using behavioural (Experiments 2a and 3a) and ERP (Experiments 2b and 3b) measures to test this effect in front-back and up-down situations, respectively. If the situational priming effect is true, we predicted that the action-evaluation effect would be modulated by front-back and up-down situations and show a reversed action-evaluation effect.

#### Participants

Another 29 undergraduate students were recruited randomly for Experiment 2a (behavioural experiment, 11 males, 20 years ± 1.8) and 24 undergraduate students were recruited for Experiment 2b (EEG experiment). In Experiment 2b, 3 participants were excluded from the data analysis due to excessive anomalies in EEG signals. Thus, 21 participants were included (9 males, 22 years ± 2.5) in the final EEG analyses. All participants provided written informed consent before the experiment. The experiment was approved by the ethical review board of the Department of Psychology, South China Normal University.

#### Materials and design

The materials and designs of Experiments 2a and 2b were the same as those in Experiment 1.

For the front-back situation in Experiments 2a and 2b, another 31 undergraduate students were recruited for subjective evaluation. They finished 20 affective valence verification trials by pushing or pulling a mouse at their right centre sides. The task was the same as that in Experiments 2a and 2b. Subsequently, the participants also selected a preferred combination from (1) push-avoidance and pull-approach; (2) push-front and pull-back; (3) push-up and pull-down; (4) other; (5) do not have special feelings. The results revealed that 27 participants (87%) chose (2), two participants (6.5%) chose (1), and two participants (6.5%) chose (5). These results indicate that most participants preferred the “push-front and pull-back” combination.

#### Procedure

The procedures for Experiment 2a and 2b were the same as those for Experiment 1, except that the mouse was placed at the right centre side of the participants. At the beginning of each trial, participants placed the mouse at their right centre sides and executed the pushing or pulling action from this location (see Fig. 4).

#### EEG recording and pre-analysis

EEG recording and data analysis methods were the same as those in Experiment 1. Incorrect trials and trials whose response times were outside ±2.5 SDs from the group mean were excluded in the following analyses. Accordingly, 6.8% of the data in Experiment 2a and 5% of the data in Experiment 2b were excluded.

At the onset of each trial in the front-back situation, a mouse was placed at the right central side of the participants. Thus, the body was the reference point for pushing and pulling: pushing is a forward action, whereas pulling is a backward action (see Figs 4 and 5). In Experiments 2a and 2b, participants also pushed or pulled the mouse from their right central sides to judge the valence of affective words. If the effect was primed differently by various situations, we would observe a “push-front-positive and pull-back-negative” association opposite to that in Experiment 1.

### Experiments 3a and 3b (the up-down situation)

#### Participants

We randomly recruited another 24 undergraduate students for Experiment 3a (behavioural experiment, 11 males, 21 years ± 2.5) and another 30 undergraduate students for Experiment 3b (EEG experiment). However, 5 participants in Experiment 3b were discarded because of excessive anomalies in EEG signals, leaving 25 participants (13 males, 22 years ± 3.1) in the following analyses. All participants provided written informed consent before the experiment. The experiment was approved by the ethical review board of the Department of Psychology, South China Normal University.

#### Materials and design

For the up-down situation in Experiments 3a and 3b,we recruited another 33 undergraduate students to participate in the subjective evaluation. They completed 20 affective verification trials by pushing or pulling the mouse at their right frontal sides. The procedure was the same as that in Experiments 3a and 3b. Next, the participants also selected a preferred combination from (1) push-avoidance and pull-approach; (2) push-front and pull-back; (3) push-up and pull-down; (4) other; (5) do not have any particular feelings. The results indicated that 29 participants (88%) chose (3), one participant (3%) chose (1), and three participants (9%) chose (5). These results suggest that the majority of participants preferred the “push-up and pull-down” combination.

The materials and design were the same as those in Experiments 2a and 2b except that the background in this experiment was replaced by an upward image (see Fig. 5, Panel B).

#### Procedure

In Experiments 3a and 3b, a mouse was located at the participants’ right frontal sides, and participants imagined they were looking at the sky. The screen was lifted 25 cm, making participants raise their heads slightly. This manipulation helped participants better focus on the upward view. To begin, a 1500-ms video was played of a gradually rising view between two buildings. After a random duration of 500–1000 ms, the target word was presented in the centre of the screen. Participants also pushed or pulled mice to verify the valence of affective words. All other aspects were the same as those in Experiments 2a and 2b.

In the up-down situation, pushing was perceived as upward and associated with positive valence, whereas pulling was perceived as downward and associated with negative valence, resulting in a “push-up-positive and pull-down-negative” association (see Fig. 4). Participants also pushed or pulled a mouse to judge the valence of affective words.

#### EEG recording and pre-analysis

EEG recording and data analysis methods were the same as those in Experiments 2a and 2b. Trials whose response times were outside ±2.5 SDs from the group mean and responses that were incorrect were discarded in the analyses. In the end, 6.5% of the data in Experiment 3a and 5.4% of the data in Experiment 3b were excluded.

## Electronic supplementary material

Supplementary Information
