# Supplementary material for: Situations restructure the congruency between action and valence in the action-evaluation effect
Source: Sci Rep. 2018 Mar 20;8:4896. doi: 10.1038/s41598-018-23095-x (PMC5861086; doi:10.1038/s41598-018-23095-x)
Supplement: Supplementary file 1 — Supplementary Information [file 41598_2018_23095_MOESM1_ESM.doc]

Supplementary Information

Situations restructure the congruency between action and valence in the action-evaluation effect

Hanlin Wang^† 1, 2 , 3, 4^, Jiushu Xie^† 1, 3 , 4^, Ce Mo^5^, Xianyou He^1, 3 , 4^, Ruiming Wang^1, 3 , 4^, Rongjun Yu^* 1, 3 , 4^, Lei Mo[[1]](#footnote-2)* 1, 3 , 4

^1^ Center for Studies of Psychological Application, South China Normal University, Guangzhou, China.

^2^ School of Education, Hebei Normal University, Shijiazhuang, China.

^3^ School of Psychology, South China Normal University, Guangzhou, China.

^4^ Guangdong Provincial Key Laboratory of Mental Health and Cognitive Science, South China Normal University, Guangzhou, China.

^5^ Peking-Tsinghua Center for Life Sciences, Academy for Advanced Interdisciplinary Studies, Peking University, Beijing, China.

To further present detailed results of behavioural and ERPs data, we split the congruency into affective valence and action in further data analyses. Then, for each experiment, we conducted 2 (positive vs. negative) × 2 (push vs. pull) ANOVAs for behavioural data and 2 (positive vs. negative) × 2 (push vs. pull) × 5 (electrode areas) ANOVAs for ERP data.

**Behavioural Response Times (RTs):**

For Experiment 1, RT data showed that the interaction between affective valence and action was significant, *F*(1,20)=6.707, *p*=.018, *η_p_*^2^=.251. Further tests showed that, for positive words, the pulling action induced faster RTs (901±192 ms) than did the pushing action (1004±238 ms), *F*(1,20)=5.657, *p*=.027, *η_p_*^2^=.220. For the negative words, the pushing action induced faster RTs (929±166 ms) than did the pulling action (1056±295 ms), *F*(1,20)=6.227, *p*=.021, *η_p_*^2^=.237.

For Experiment 2a, RTs results showed that the interaction between affective valence and action was significant, *F*(1,28)=5.336, *p*=.028, *η_p_*^2^=.160. Further tests showed that, for positive words, the pushing action induced faster RTs (740±134 ms) than did the pulling action (801±147 ms), *F*(1,28)=12.801, *p*=.001, *η_p_*^2^=.314. For negative words, no significant difference between pulling and pushing actions was observed, *F*(1,28)=.615, *p*=.439.

For Experiment 2b, RTs results showed that the interaction between affective valence and action was significant, *F*(1,20)=7.218, *p*=.014, *η_p_*^2^=.265. Further tests showed that, for positive words, the pushing action induced faster RTs (737±153 ms) than did the pulling action (786±148 ms), *F*(1,20)=6.345, *p*=.020, *η_p_*^2^=.241. For negative words, no such effect was found, *F*(1,20)=3.540, *p*=.075.

For Experiment 3a, RTs results showed that the interaction between affective valence and action was significant, *F*(1,23)=5.142, *p*=.033, *η_p_*^2^=.183. Further tests showed that, for positive words, the pushing action induced faster RTs (706±130 ms) than did the pulling action (765±170 ms), *F*(1,23)=10.615, *p*=.003, *η_p_*^2^=.316. For negative words, such effect was not observed, *F*(1,23)=1.046, *p*=.317.

For Experiment 3b, RTs results showed that the interaction between affective valence and action was significant, *F*(1,24)=10.300, *p*=.004, *η_p_*^2^=.300. Further tests showed that, for positive words, the pushing action induced faster RTs (781±147 ms) than did the pulling action (832±160 ms), *F*(1,24)=15.617, *p*=.001, *η_p_*^2^=.394. For negative words, no such effect was observed, *F*(1,24)=2.802, *p*=.107 (See Table 1).

Supplementary Table S1. Three-way ANOVA results based on RTs of 3 experiments

|  |  |  | *F* | *p* | *η^2^* |
| --- | --- | --- | --- | --- | --- |
| Far-Near | Experiment 1 | Valence | 7.417 | .013 | .271 |
|  |  | Action | .524 | .478 | .026 |
|  |  | Valence×Action | 6.707 | .018 | .251 |
| Front-Behind | Experiment 2a | Valence | 23.483 | <.001 | .456 |
|  |  | Action | 4.676 | .039 | .143 |
|  |  | Valence×Action | 5.336 | .028 | .16 |
|  | Experiment 2b | Valence | 16.48 | .001 | .452 |
|  |  | Action | .001 | .982 | <.001 |
|  |  | Valence×Action | 7.218 | .014 | .265 |
| Up-Down | Experiment 3a | Valence | 8.373 | .008 | .267 |
|  |  | Action | 4.568 | .043 | .166 |
|  |  | Valence×Action | 5.142 | .033 | .183 |
|  | Experiment 3b | Valence | 21.88 | <.001 | .477 |
|  |  | Action | 1.271 | .271 | .05 |
|  |  | Valence×Action | 10.3 | .004 | .3 |

**Event-Related Potentials (ERP) results:**

For Experiment 1, the results showed that the interaction between affective valence and action was significant, *F*(1,20)=4.912, *p*=.038, *η_p_*^2^=.197. Further tests showed that, for positive words, the pulling action induced a smaller P2 component (4.744±.715 μV) than did the pushing action (5.242±.708 μV). This difference reached marginal significance, *F*(1,20)=3.818, *p*=.065, *η_p_*^2^=.160. For negative words, no significant differences between pulling and pushing actions was observed, *F*(1,20)=1.884, *p*=.185.

For Experiment 2b, the interaction between affective valence and action was significant, *F*(1,20)=4.659, *p*=.043, *η_p_*^2^=.189. Further tests showed that, for positive words, the pulling action induced a larger P2 component (3.193±.599 μV) than did the pushing action (2.709±.649 μV) and this difference only reached marginal significance, *F*(1,20)=3.694, *p*=.069, *η_p_*^2^=.156. For negative words, no significant differences between pulling and pushing actions was observed, *F*(1,20)=1.764, *p*=.199.

For Experiment 3b, the interaction between affective valence, action, and electrode site was significant, *F*(4,96)=8.165, *p*=.005, *η_p_*^2^=.254. Further tests showed that, for positive words, the pulling action induced a larger P2 component than did the pushing action in the central-parietal and parietal areas, *F*central-parietal (1,24)=4.777, *p*=.039, *η_p_*^2^=.166, *F*parietal (1,24)=8.735, *p*=.007, *η_p_*2=.267. For negative words, the pulling action induced a smaller P2 than did the pushing action in the parietal areas, *F*parietal (1,24)=7.818, *p*=.010, *η_p_*^2^=.246 (see Table 2).

In general, these results revealed that positive words could be more sensitively affected by actions than negative words. This could be interpreted by the polarity correspondence account (Lakens, 2012), which proposes that the processing of + polar (e.g., positive words), but not – polar (e.g., negative words), could benefit from the congruent effect.

Supplementary Table S2. Three-way ANOVA results based on mean amplitude of 3 experiments.

|  | Experiment 1  Far-Near Situation | | | Experiment 2b  Front-Behind Situation | | | Experiment 3b  Up-Down Situation | | |
| --- | --- | --- | --- | --- | --- | --- | --- | --- | --- |
|  | *F* | *p* | *η_p_^2^* | *F* | *p* | *η_p_^2^* | *F* | *p* | *η_p_^2^* |
| Valence | .001 | n.s. |  | .295 | n.s. |  | .28 | n.s. |  |
| Action | .025 | n.s. |  | .512 | n.s. |  | .529 | n.s. |  |
| Electrode Site | 3.626 | n.s. |  | 5.56 | .022 | .218 | 1.197 | n.s. |  |
| Valence×Action | 4.912 | .038 | .197 | 4.659 | .043 | .189 | 1.027 | n.s. |  |
| Valence×Electrode Site | .433 | n.s. |  | 1.393 | n.s. |  | 2.515 | n.s. |  |
| Valence×Electrode Site | 3.777 | .049 | .159 | .201 | n.s. |  | 1.108 | n.s. |  |
| Valence×Action×Electrode Site | .714 | n.s. |  | .338 | n.s. |  | 8.165 | .005 | .254 |

Supplementary Table S3. The repeated-measure ANOVA results of N1 in 3 experiments^*^

|  | Experiment 1:  Far-Near (Time Window: 200-300 ms) | | | Experiment 2:  Front-Behind (Time Window: 80-130 ms) | | | Experiment 3:  Up-Down (Time Window: 100-150 ms) | | |
| --- | --- | --- | --- | --- | --- | --- | --- | --- | --- |
|  | *F* | *p* | *η_p_^2^* | *F* | *p* | *η_p_^2^* | *F* | *p* | *η_p_^2^* |
| Condition | 3.518 | n.s. |  | .063 | n.s. |  | .96 | n.s. |  |
| Areas | 5.736 | .021 | .223 | 13.941 | .001 | .411 | 10.799 | .002 | .31 |
| Condition×Areas | .173 | n.s. |  | 1.037 | n.s. |  | 3.556 | n.s. |  |

^*^Three 2 (condition: congruent vs. incongruent) × 5 (electrode areas: frontal, frontal-central, central, central-parietal, and partial) repeated-measure ANOVAs were conducted.

1. ^^† These authors contributed equally to this work and are co-first authors.

   ^*^ Corresponding: Lei Mo, Center for Studies of Psychological Application, School of Psychology, South China Normal University, Guangzhou 510631, China (Email: molei@scnu.edu.cn) or Rongjun Yu, Center for Studies of Psychological Application, School of Psychology, South China Normal University, Guangzhou 510631, China (E-mail: rongjun.yu@gmail.com). [↑](#footnote-ref-2)
